# Supplementary figures and images for: A secretory hexokinase plays an active role in the proliferation of Nosema bombycis
Source: PeerJ. 2018 Sep 21;6:e5658. doi: 10.7717/peerj.5658 (PMC6152459; doi:10.7717/peerj.5658)

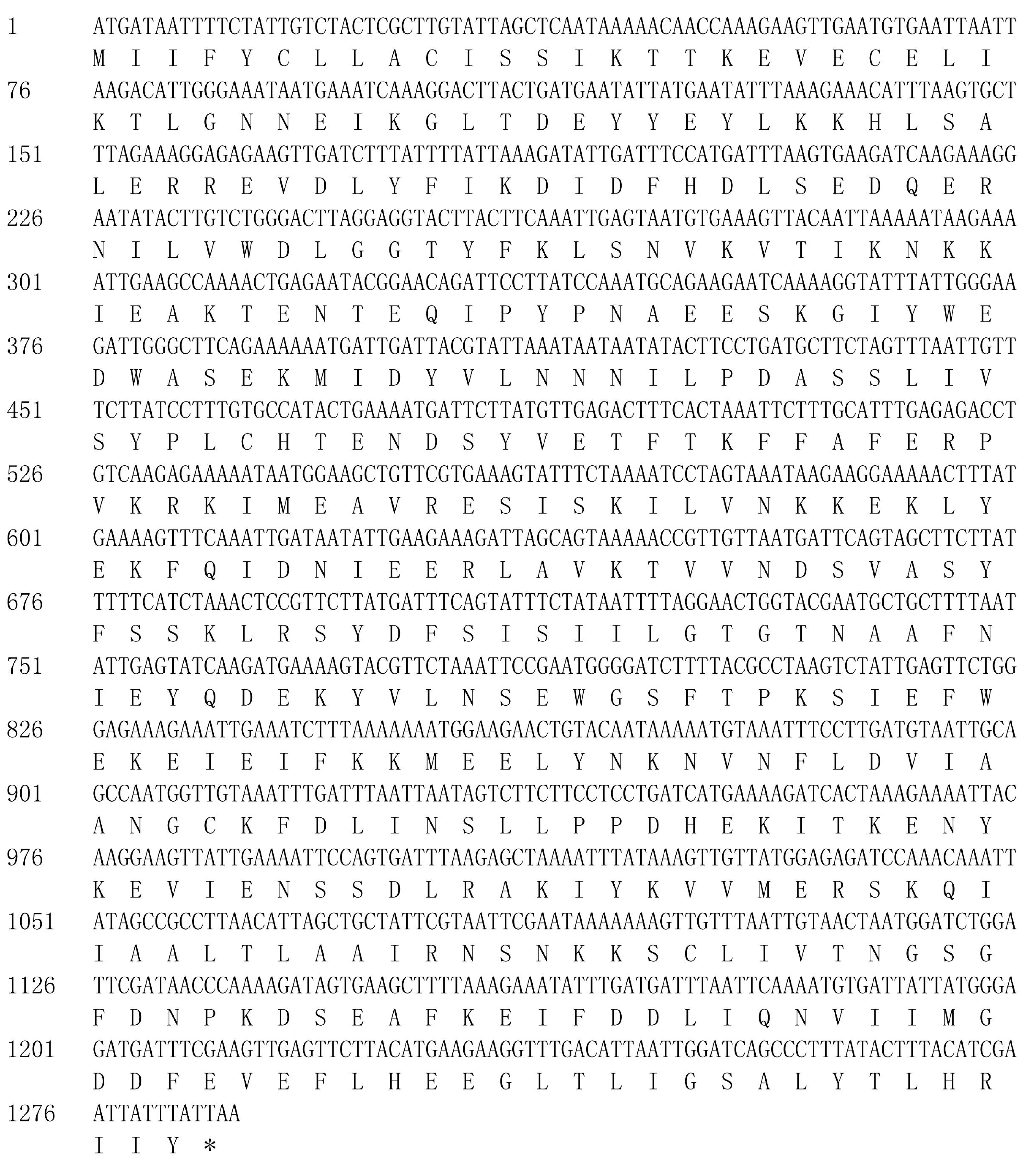

Supplement: Figure S1 — The NbHK sequencing results showed a 1,287-bp fragment encoding 428 amino acids. [file peerj-06-5658-s001.png]

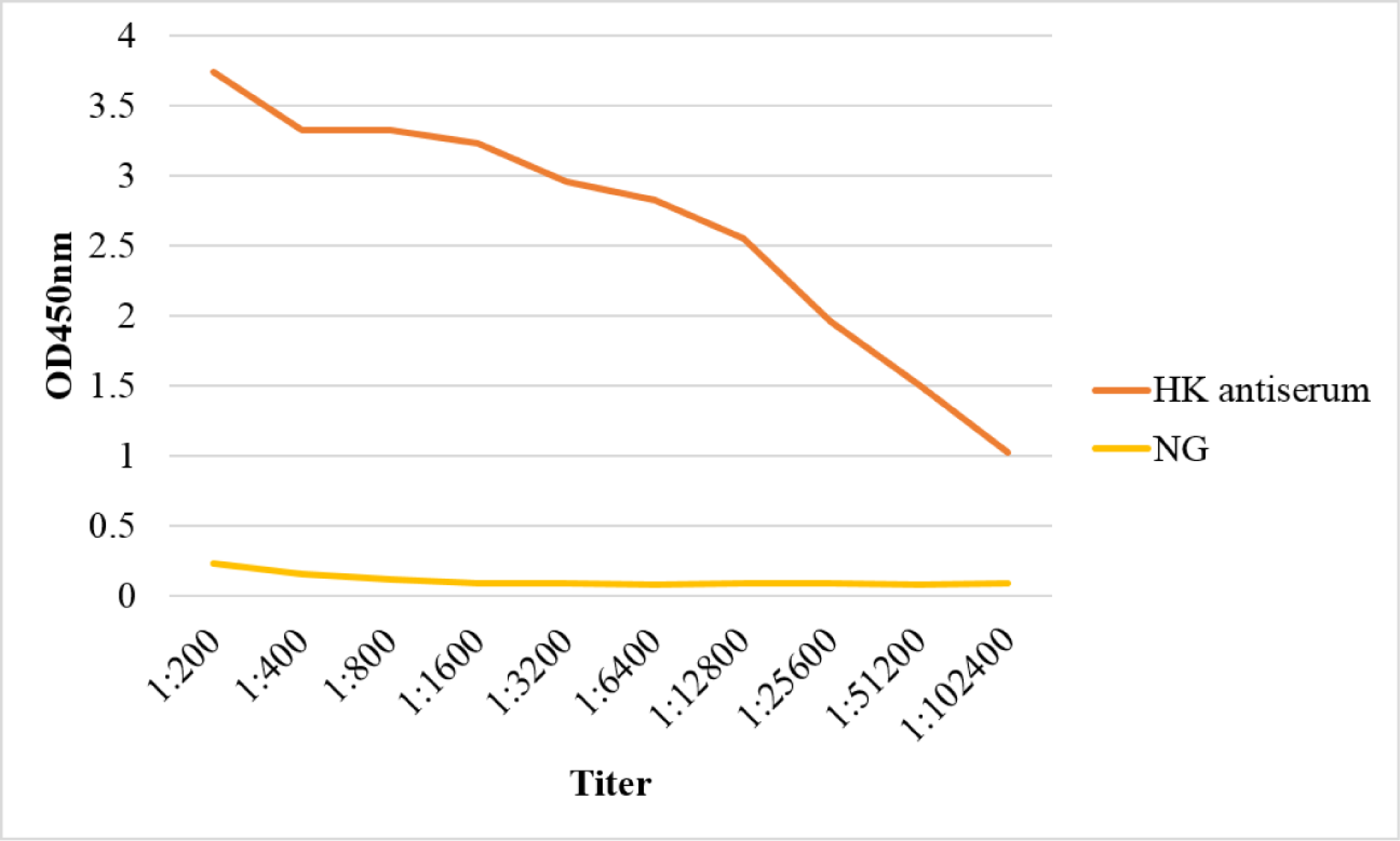

Supplement: Figure S2 — The titer of HK antiserum was detected by ELISA. Titer of unimmunized BALB/c mouse was as the negative control. The result of ELISA showed the titer of HK antibody was 1:102400. [file peerj-06-5658-s002.png]

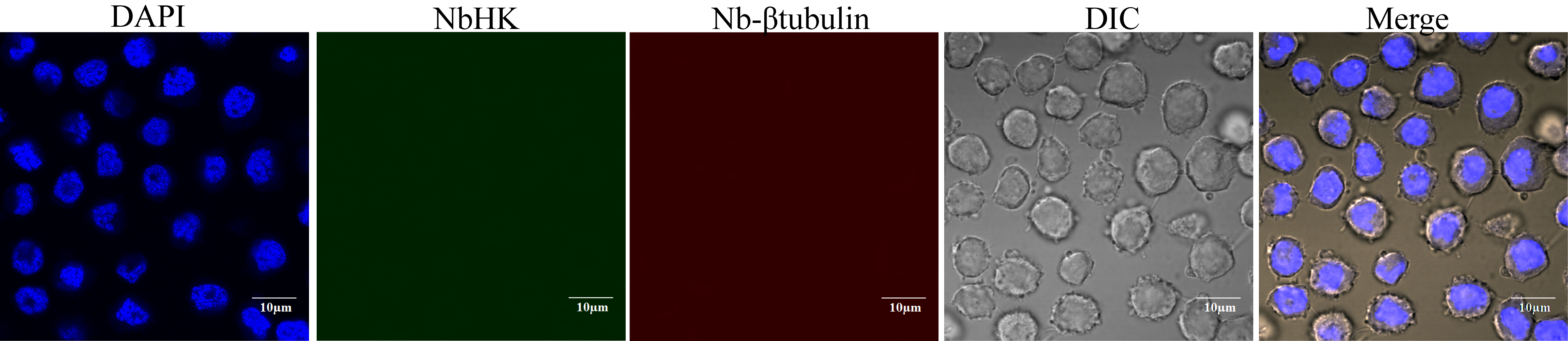

Supplement: Figure S3 — Healthy Sf9-III cells were used to detect specificity of Nbβ-tubulin and NbHK antiserum. Cell nuclei were label with DAPI (blue). Binding of Nbβ-tubulin and NbHK were detect with Alexa 594 and 488 respectively. There were no any signals of Nbβ-tubulin and NbHK in healthy Sf9-III cells ’nuclei and cytoplasm. [file peerj-06-5658-s003.png]

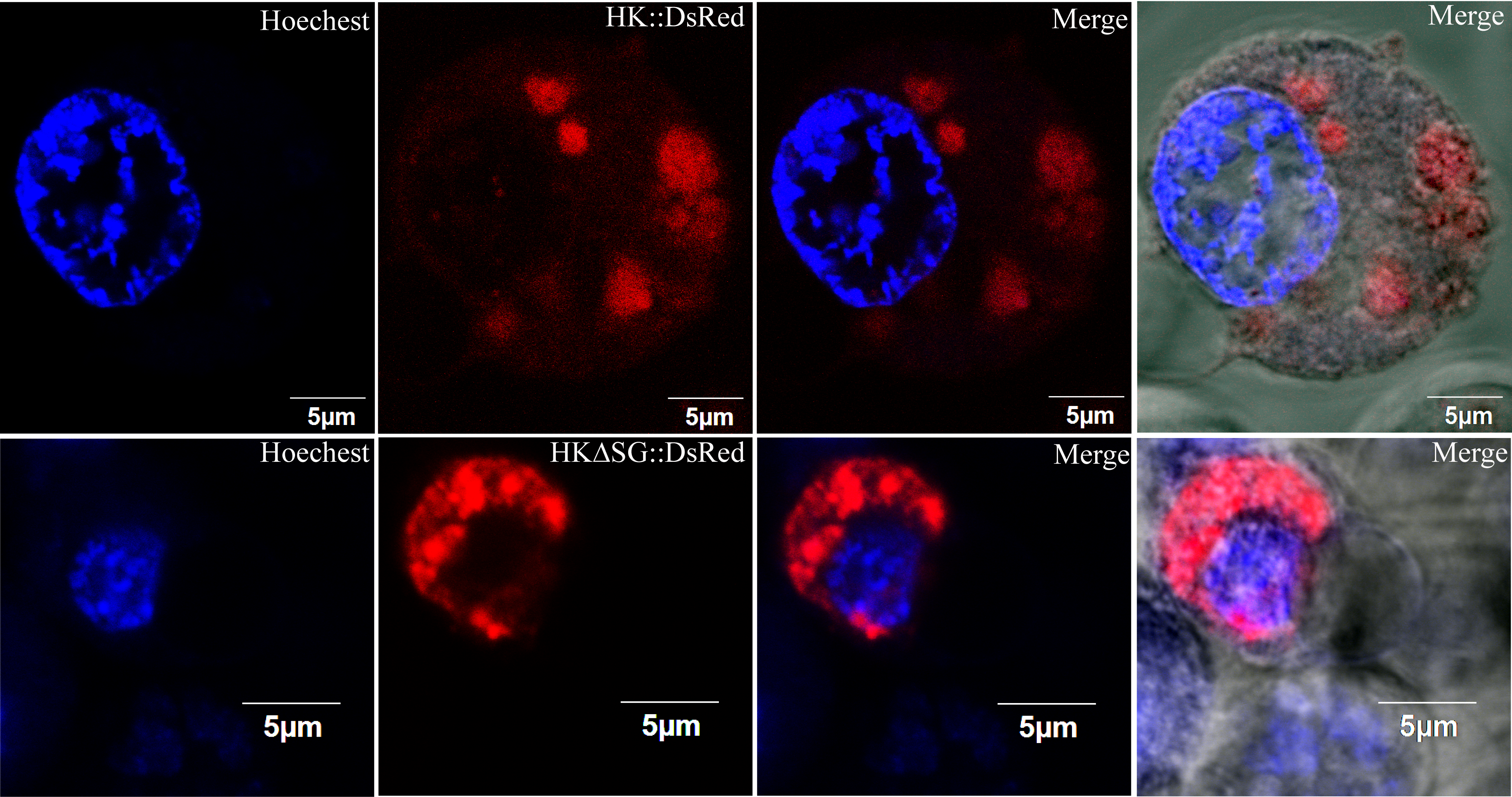

Supplement: Figure S4 — HK with and without signal peptide fusing DsRed were located in cytoplasm. [file peerj-06-5658-s004.png]

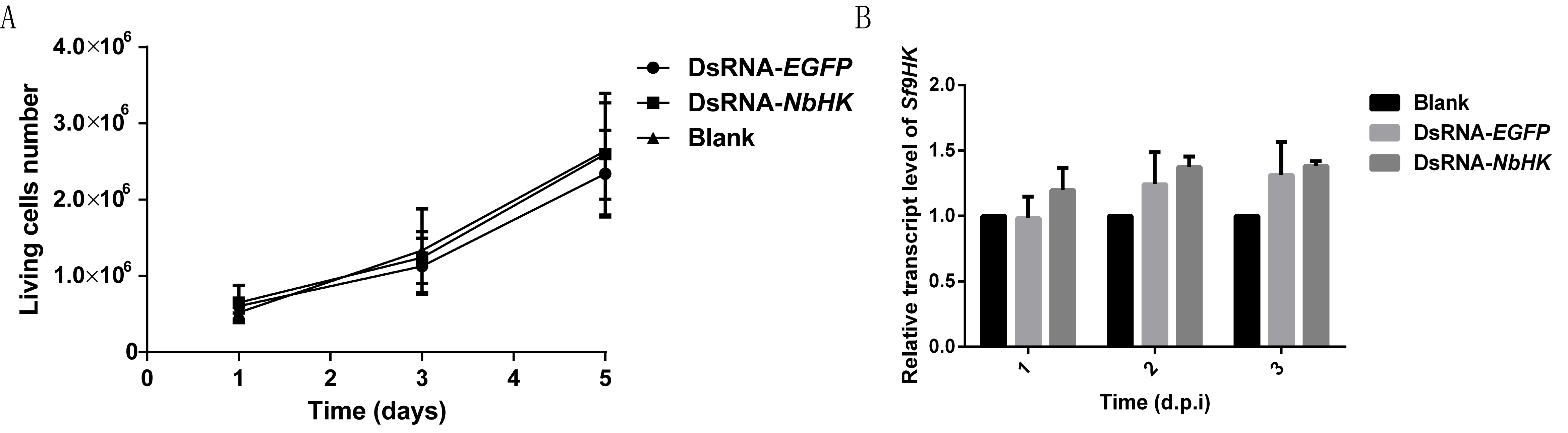

Supplement: Figure S5 — Quantity PCR and live cell counting were used to detect the impact of dsRNA-EGFP or dsRNA-NbHK on host cells. Equal numbers of Sf9-III cells were transiently transfected with dsRNA-EGFP or dsRNA-NbHK. Blank group was without any dsRNA. (A) The impact of RNAi constructs on cell proliferation. Ten microliters cell suspension samples were mixed isopycnic trypan blue, and then the living cells were detected by Cell Count. (B) The transcription levels of Sf9-III hexokinase. Complementary DNAs of the blank, experimental (dsRNA-NbHK) and mock (dsRNA-EGFP) groups were analyzed by qPCR. GAPDH of Sf9-III was reference gene to normalize samples. The △△Ct method was conduct to process the data. Vertical bars show the mean ±SEs (n = 3). [file peerj-06-5658-s005.png]

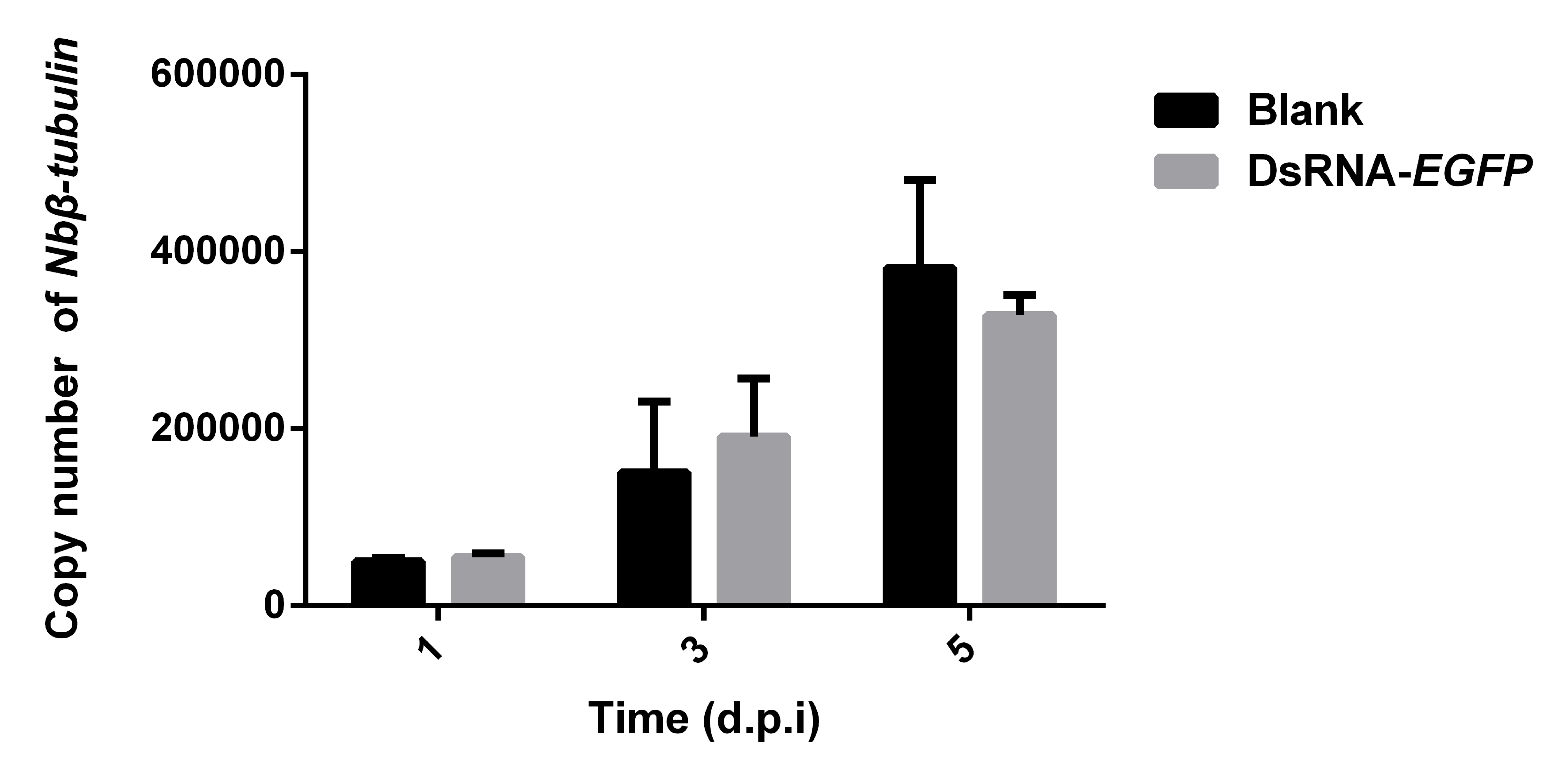

Supplement: Figure S6 — N. bombycis proliferation in Sf9-III cells. Genomic DNA was extracted from blank and mock (dsRNA-EGFP) groups at 1, 3 and 5 d.p.i. Copy numbers of Nbβ-tubulin indicated the proliferation of N. bombycis. Vertical bars show the mean ± SEs (n = 3). [file peerj-06-5658-s006.png]

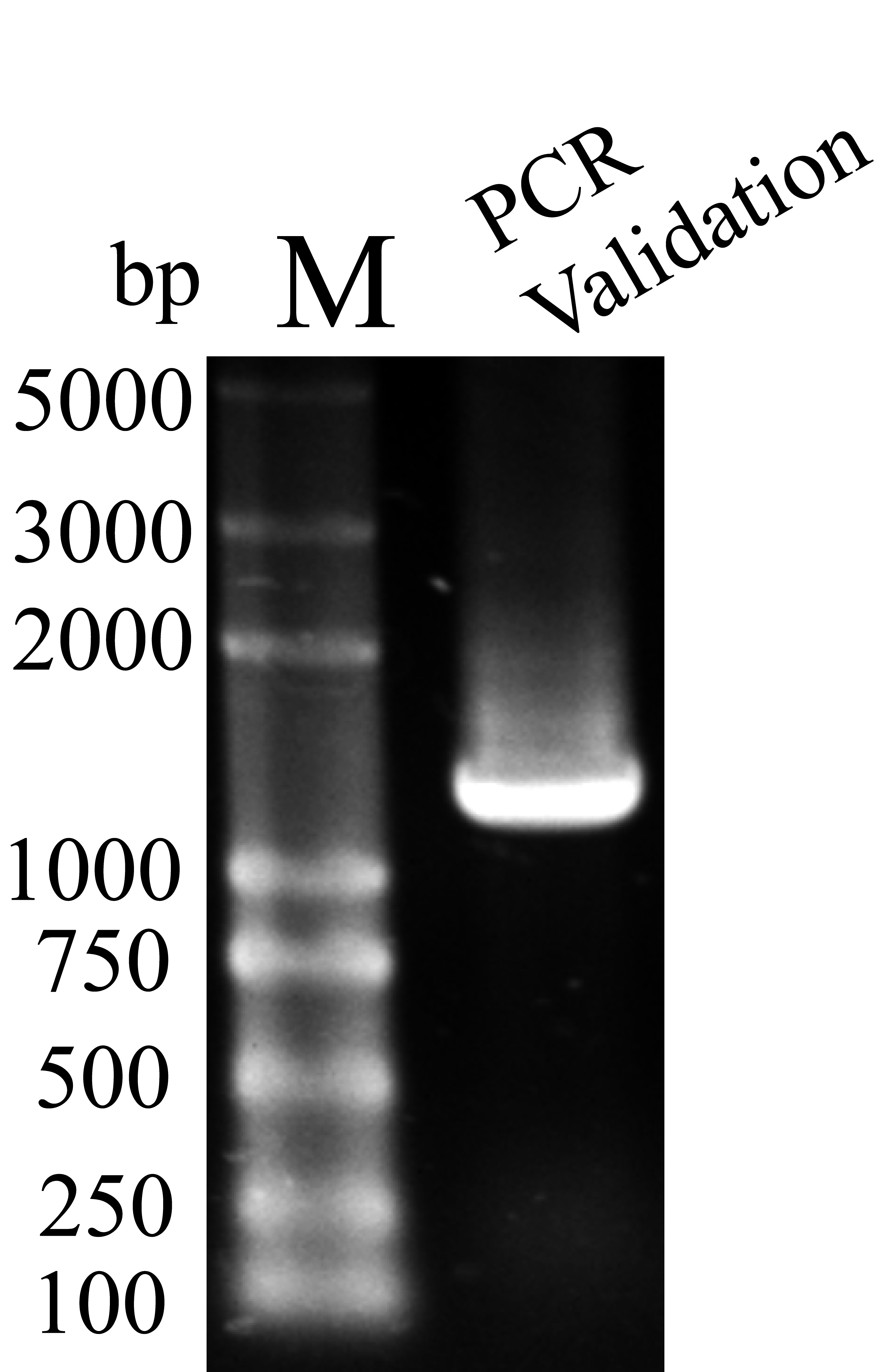

Supplement: Data S1 — The full-length electrophoretic gels and western blot of Fig. 1. [file peerj-06-5658-s011.zip › Supplemental Data S1/Figure 1 B left.png]

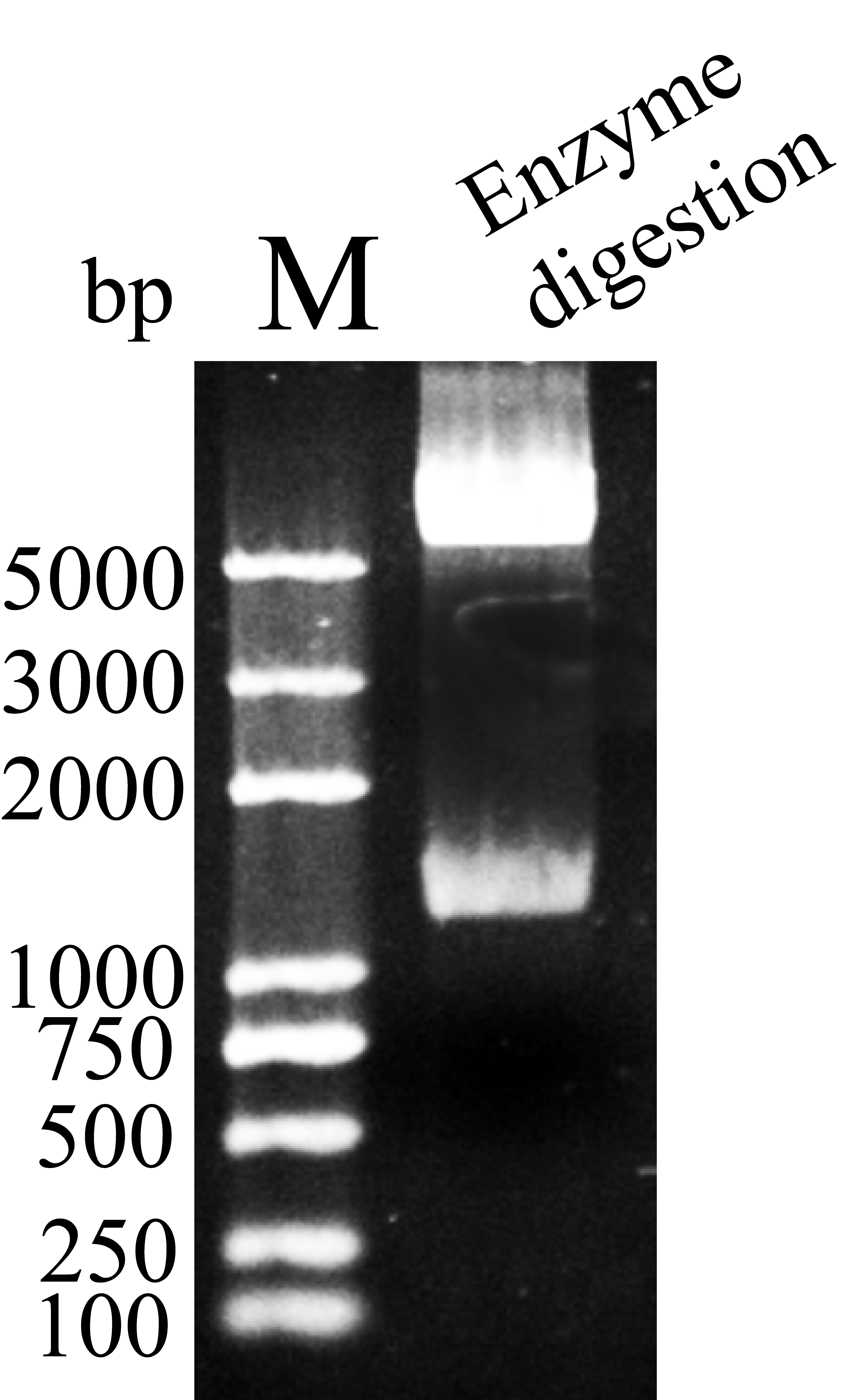

Supplement: Data S1 — The full-length electrophoretic gels and western blot of Fig. 1. [file peerj-06-5658-s011.zip › Supplemental Data S1/Figure 1 B right.png]

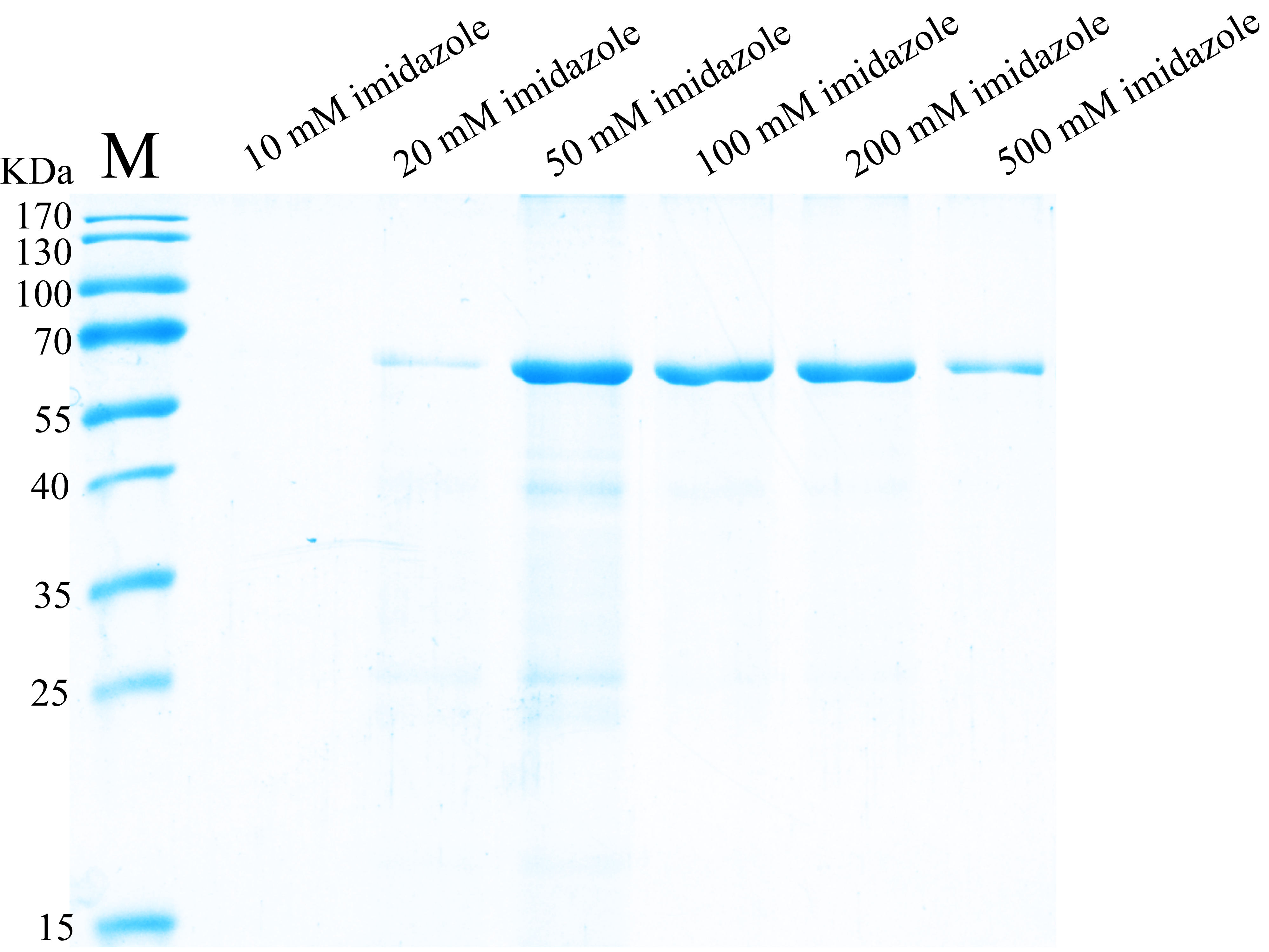

Supplement: Data S1 — The full-length electrophoretic gels and western blot of Fig. 1. [file peerj-06-5658-s011.zip › Supplemental Data S1/Figure 1 C.png]

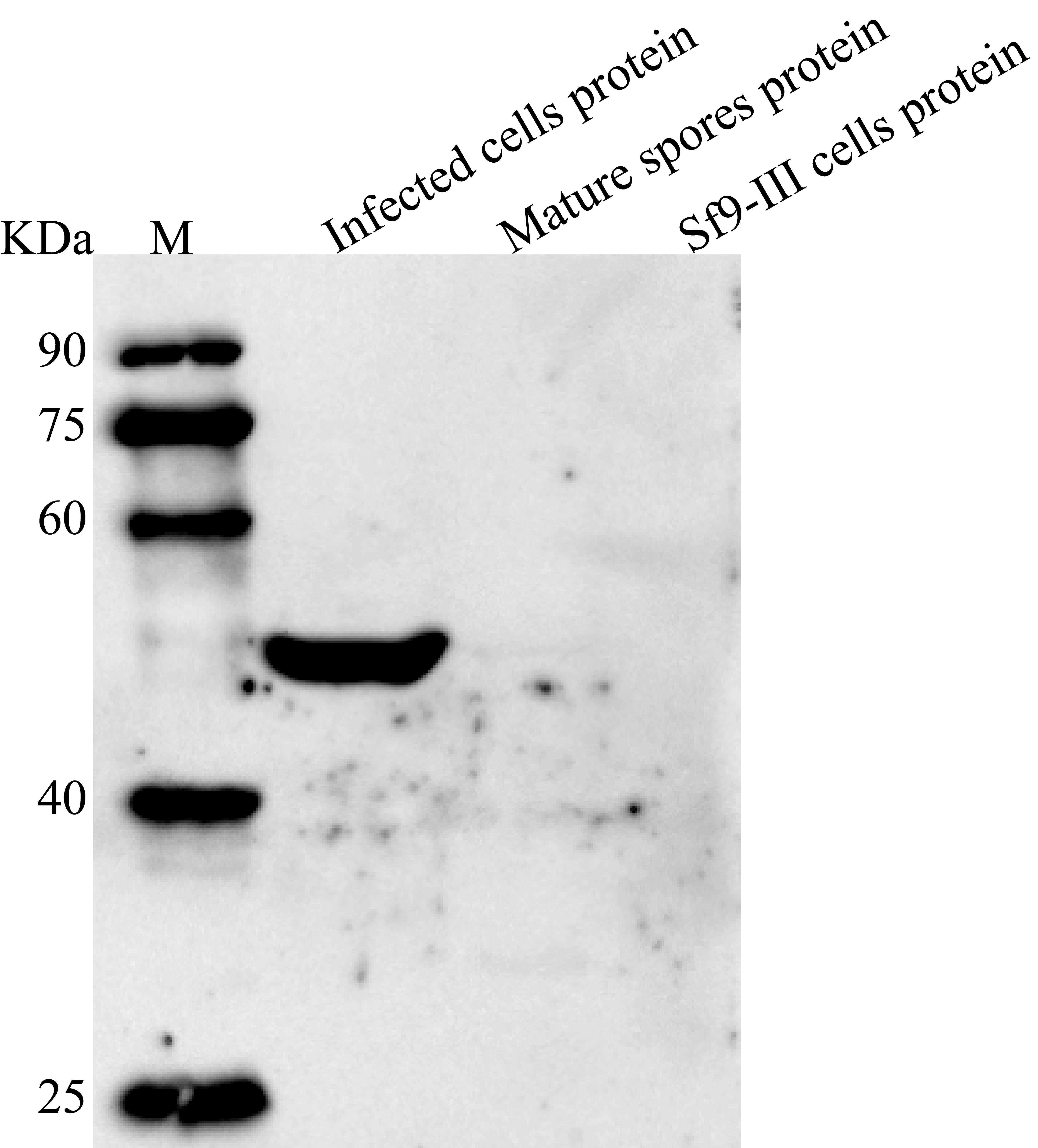

Supplement: Data S1 — The full-length electrophoretic gels and western blot of Fig. 1. [file peerj-06-5658-s011.zip › Supplemental Data S1/Figure 1 D.png]

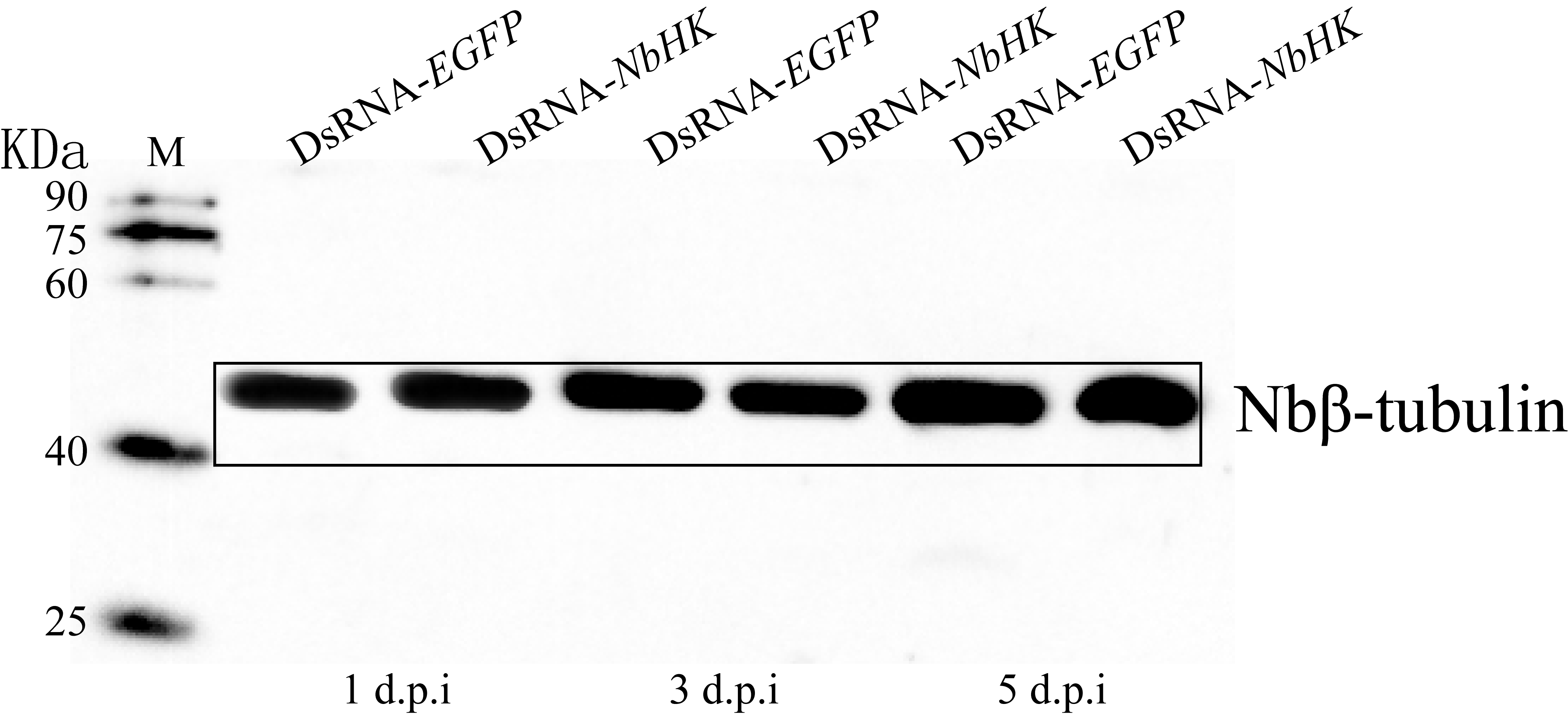

Supplement: Data S2 — The full-length western blot of Fig. 5B. [file peerj-06-5658-s012.zip › Supplemental Data S2/Fiure 5 B (down).png]

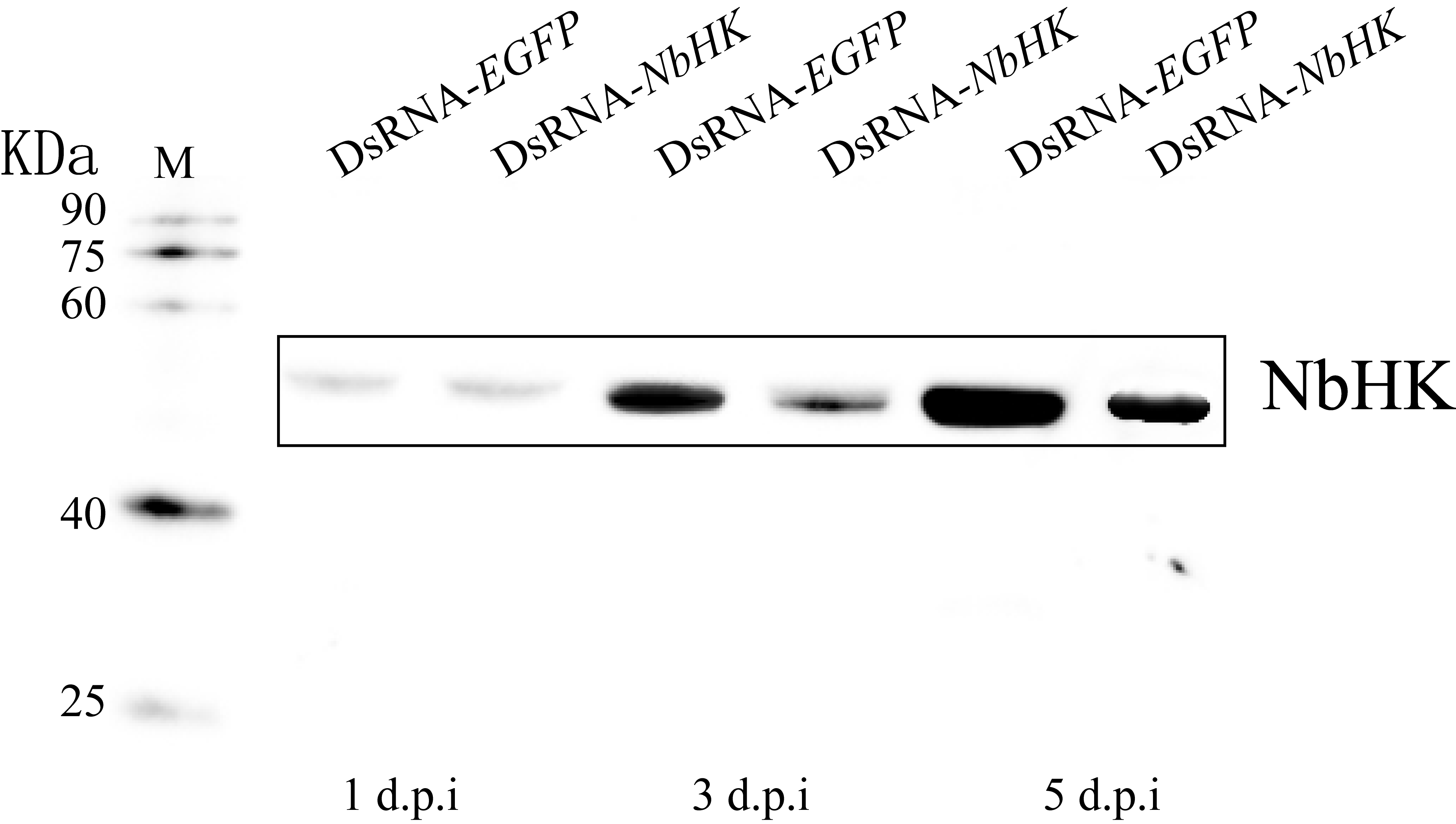

Supplement: Data S2 — The full-length western blot of Fig. 5B. [file peerj-06-5658-s012.zip › Supplemental Data S2/Fiure 5 B (upper).png]

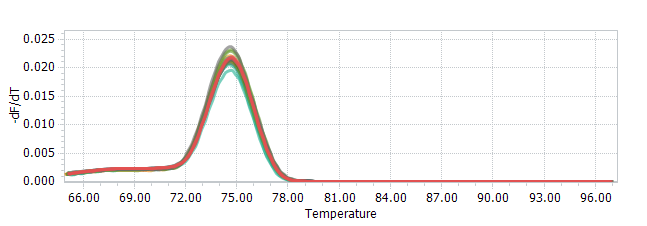

Supplement: Data S3 — SYBR Green was used in all qPCR. Melting curve of NbHK, NbSSU, Nb β-tubulin, SfGAPDH and SfHK demonstrate the amplifications were specific. [file peerj-06-5658-s013.zip › Supplemental Data S3/NbHK.png]

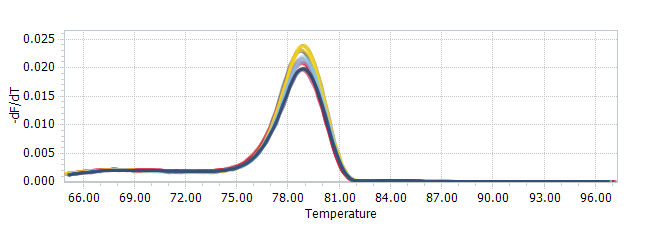

Supplement: Data S3 — SYBR Green was used in all qPCR. Melting curve of NbHK, NbSSU, Nb β-tubulin, SfGAPDH and SfHK demonstrate the amplifications were specific. [file peerj-06-5658-s013.zip › Supplemental Data S3/NbSSU.png]

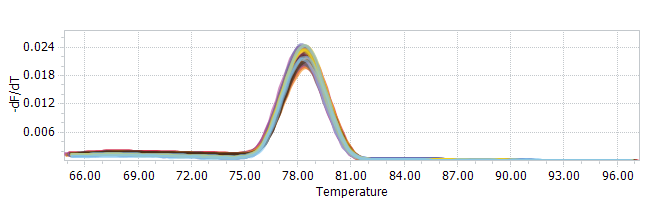

Supplement: Data S3 — SYBR Green was used in all qPCR. Melting curve of NbHK, NbSSU, Nb β-tubulin, SfGAPDH and SfHK demonstrate the amplifications were specific. [file peerj-06-5658-s013.zip › Supplemental Data S3/Nba┬-tubulin.png]

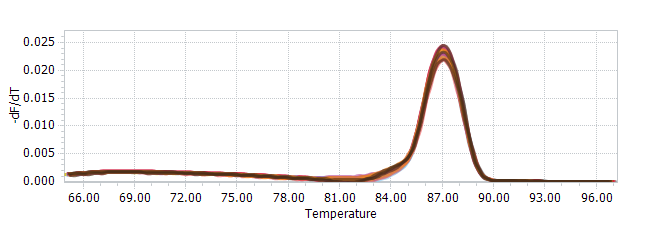

Supplement: Data S3 — SYBR Green was used in all qPCR. Melting curve of NbHK, NbSSU, Nb β-tubulin, SfGAPDH and SfHK demonstrate the amplifications were specific. [file peerj-06-5658-s013.zip › Supplemental Data S3/SfGAPDH.png]

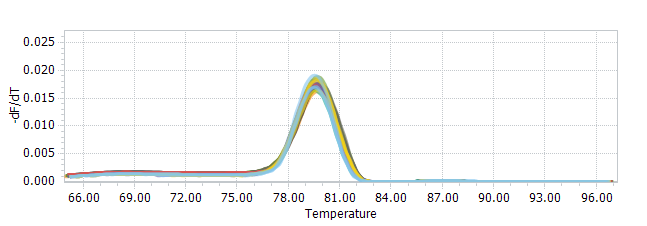

Supplement: Data S3 — SYBR Green was used in all qPCR. Melting curve of NbHK, NbSSU, Nb β-tubulin, SfGAPDH and SfHK demonstrate the amplifications were specific. [file peerj-06-5658-s013.zip › Supplemental Data S3/SfHK.png]
